# Synergistic Association of Hepatitis B Surface Antigen and Plasma Epstein-Barr Virus DNA Load on Distant Metastasis in Patients With Nasopharyngeal Carcinoma

**DOI:** 10.1001/jamanetworkopen.2022.53832

**Published:** 2023-02-09

**Authors:** Haojiang Li, Di Cao, Shuqi Li, Binghong Chen, Yun Zhang, Yuliang Zhu, Chao Luo, Weiqun Lin, Wenjie Huang, Guangying Ruan, Rong Zhang, Jiang Li, Lizhi Liu

**Affiliations:** 1 Department of Radiology, Sun Yat-sen University Cancer Center, State Key Laboratory of Oncology in South China, Collaborative Innovation Center for Cancer Medicine, Guangdong Key Laboratory of Nasopharyngeal Carcinoma Diagnosis and Therapy, Guangzhou, China; 2 Department of Endoscopy, Sun Yat-sen University Cancer Center, State Key Laboratory of Oncology in South China, Collaborative Innovation Center for Cancer Medicine, Guangdong Key Laboratory of Nasopharyngeal Carcinoma Diagnosis and Therapy, Guangzhou, China; 3 Department of Endoscopy, National Cancer Center/National Clinical Research Center for Cancer/Cancer Hospital & Shenzhen Hospital, Chinese Academy of Medical Sciences and Peking Union Medical College, Shenzhen, China; 4 Nasopharyngeal Head and Neck Tumor Radiotherapy Department, Zhongshan City People’s Hospital, Zhongshan, China; 5 Department of Clinical Nutrition, The First Affiliated Hospital of Guangdong Pharmaceutical University, Guangzhou, China; 6 Department of Biotherapy, Sun Yat-sen University Cancer Center, State Key Laboratory of Oncology in South China, Collaborative Innovation Center for Cancer Medicine, Guangdong Key Laboratory of Nasopharyngeal Carcinoma Diagnosis and Therapy, Guangzhou, China; 7 Department of Radiology, The Third People’s Hospital of Shenzhen, Shenzhen, China

## Abstract

**Importance:**

Hepatitis B surface antigen (HBsAg) reportedly increases the risk of distant metastasis among patients with nasopharyngeal carcinoma (NPC). However, the associated potential interaction and changes in hazard ratios (HRs) between HBsAg and different plasma Epstein-Barr (EBV) DNA levels are unknown. Moreover, the potential HBsAg-positive–associated NPC metastatic mechanism remains unclear.

**Objective:**

To investigate the prognostic value and biological associations of HBsAg and plasma EBV DNA levels on distant metastasis in patients with NPC.

**Design, Setting, and Participants:**

Retrospective cohort study performed at Sun Yat-sen University Cancer Center between January 2010 and January 2013. A total of 792 patients with nonmetastatic NPC were enrolled. The median (range) follow-up time was 62.1 (1.4-83.4) months. Of these patients, 17.8% presented with HBsAg positivity. Cytological experiments were performed to evaluate the role of HBsAg in the invasion and migration of EBV-positive NPC cells. Data analysis was performed from July 2020 to April 2021.

**Main Outcomes and Measures:**

The primary end point was distant metastasis–free survival. Association rules were used to identify new rules related to distant metastasis. Interaction plots, univariate and multivariate Cox regression analyses, stratification analysis, and quantification using HRs were conducted. Additionally, cell migration and invasion assays, as well as Western blotting, were performed in the cytological validation.

**Results:**

Among the 792 patients, 576 (72.7%) were male, with a median (IQR) age of 45 (38-53) years. The HBsAg-positive group exhibited a significant interaction and increased risk of distant metastasis when plasma EBV DNA cutoff levels were 1.5 × 1000 copies/mL or greater. The HR was 9.16 (95% CI, 2.46-34.14) when the plasma EBV DNA load reached 6 × 1000 copies/mL, which was higher than that in patients with stage IV disease (HR, 2.01; 95% CI, 1.13-3.56; *P* = .02). In cytological experiments, HBsAg promoted epithelial-mesenchymal transition by upregulating vimentin and fibronectin in EBV-positive NPC cells in vitro, thereby promoting invasion and migration of EBV-positive NPC cells.

**Conclusions and Relevance:**

In this cohort study, the observed synergistic association between HBsAg and plasma EBV DNA load represented a novel potential mechanism underlying the increased risk of distant metastasis in patients with NPC. Hence, attention should be paid to patients with NPC with HBsAg positivity, especially when the plasma EBV DNA level is 6 × 1000 copies/mL or greater. Consideration of this synergistic association will contribute to more accurate individualized management.

## Key Points

**Question:**

Is hepatitis B surface antigen (HBsAg) associated with the prognostic value of plasma Epstein-Barr virus (EBV) DNA on distant metastasis among patients with nasopharyngeal carcinoma (NPC)?

**Findings:**

In this cohort study of 792 patients with NPC, increased risk of metastasis occurred in HBsAg-positive patients when plasma EBV DNA level was 1.5 × 1000 copies/mL or greater compared with HBsAg-negative patients. In cytological experiments, HBsAg promoted epithelial-mesenchymal transition in EBV-positive NPC cells, which may account for the high metastasis rate.

**Meaning:**

The synergistic association between HBsAg and plasma EBV DNA load should be considered for the individualized management for patients with NPC due to its potential exacerbating role in metastatic risk.

## Teaser

This cohort study investigates the prognostic value and biological associations of hepatitis B surface antigen and plasma Epstein-Barr virus DNA levels with distant metastasis in patients with nasopharyngeal carcinoma.

## Introduction

Distant metastasis is a major concern in nasopharyngeal carcinoma (NPC), with the highest percentage (11.9%) among all failure patterns reported,^1^ despite considerably improved local control through multimodality management strategies.^2,3,4,5,6^ Plasma Epstein-Barr virus (EBV) DNA level is associated with tumor burden^7,8^ and treatment response in NPC^9,10^ and is independently associated with distant metastasis.^11,12,13^ Patients with NPC with high plasma EBV DNA load are more susceptible to distant metastasis^14,15^; however, the appropriate cutoff value for risk stratification remains undetermined.^11,12,13,16,17,18^

Concerns regarding hepatitis B virus (HBV) infection^19^ in NPC are increasing owing to the high rates of incidence and distant metastasis.^20,21,22^ Hepatitis B surface antigen (HBsAg) is an independent risk factor in patients with early-stage NPC; its presence is associated with a 3.7-fold higher likelihood of distant metastasis.^21^ However, the pretreatment plasma EBV DNA level, a distant metastasis biomarker, is not a confounding factor. Considering that patients positive for EBV and HBsAg exhibit significantly lower distant metastasis–free survival (DMFS),^22^ potential EBV-HBsAg interactions may exist.^22^

Different metastatic mechanisms in NPC with HBV infection have been proposed. Inflammatory infiltrating lymphocytes^22,23^ and host immune system impairment^20,21^ caused by HBV and EBV infection create a metastatic microenvironment. Meanwhile, NPC progression with EBV/HBV coinfection has been attributed to the independent role of EBV rather than the interplay between the 2 viruses.^22^ We previously reported that although induction chemotherapy eliminates micrometastasis, it leads to worse DMFS and progression-free survival in patients with NPC with HBsAg positivity.^24^ Chemotherapy further suppresses immunity, leading to HBV reactivation and increased incidence of severe hepatitis in patients with cancer and HBV infection,^25,26,27^ reducing the overall survival; however, this factor does not adequately explain the high distant metastasis rate.

Epithelial-mesenchymal transition (EMT) is essential in tumor metastasis. EBV-encoded proteins such as Epstein-Barr virus nuclear antigen 1 and latent membrane proteins can induce EMT,^23,28,29,30^ thereby promoting distant metastasis in patients with NPC. The HBV X protein reportedly triggers EMT in NPC cells by upregulating yes-associated protein 1.^31^ Moreover, small HBsAg proteins induce EMT in HBV-related hepatocellular carcinoma cells.^32^ However, whether HBsAg can influence EMT in NPC cells remains unknown.

The current retrospective cohort study investigated the prognostic value, biological associations, and associations of HBsAg and plasma EBV DNA levels with distant metastasis in NPC. Additionally, Boyden chamber assays and Western blotting with EMT-related markers were performed to investigate the role of HBsAg in EBV-negative or EBV-positive NPC cells.

## Methods

### Study Design and Ethics Approval

This retrospective clinical study comprised 792 patients with NPC treated at Sun Yat-sen University Cancer Center (SYSUCC), and cytological experiments including NPC cell migration and invasion assays and Western blot analyses were performed. Ethics approval was obtained from the Institutional Review Board of SYSUCC (approval number: B2019-222-01). Owing to the retrospective nature of the study, the requirement for informed consent was waived under the tenets of the Declaration of Helsinki. The study followed the Strengthening the Reporting of Observational Studies in Epidemiology (STROBE) reporting guidelines.

### Clinical Research Design

#### Patients

**Figure 1.**
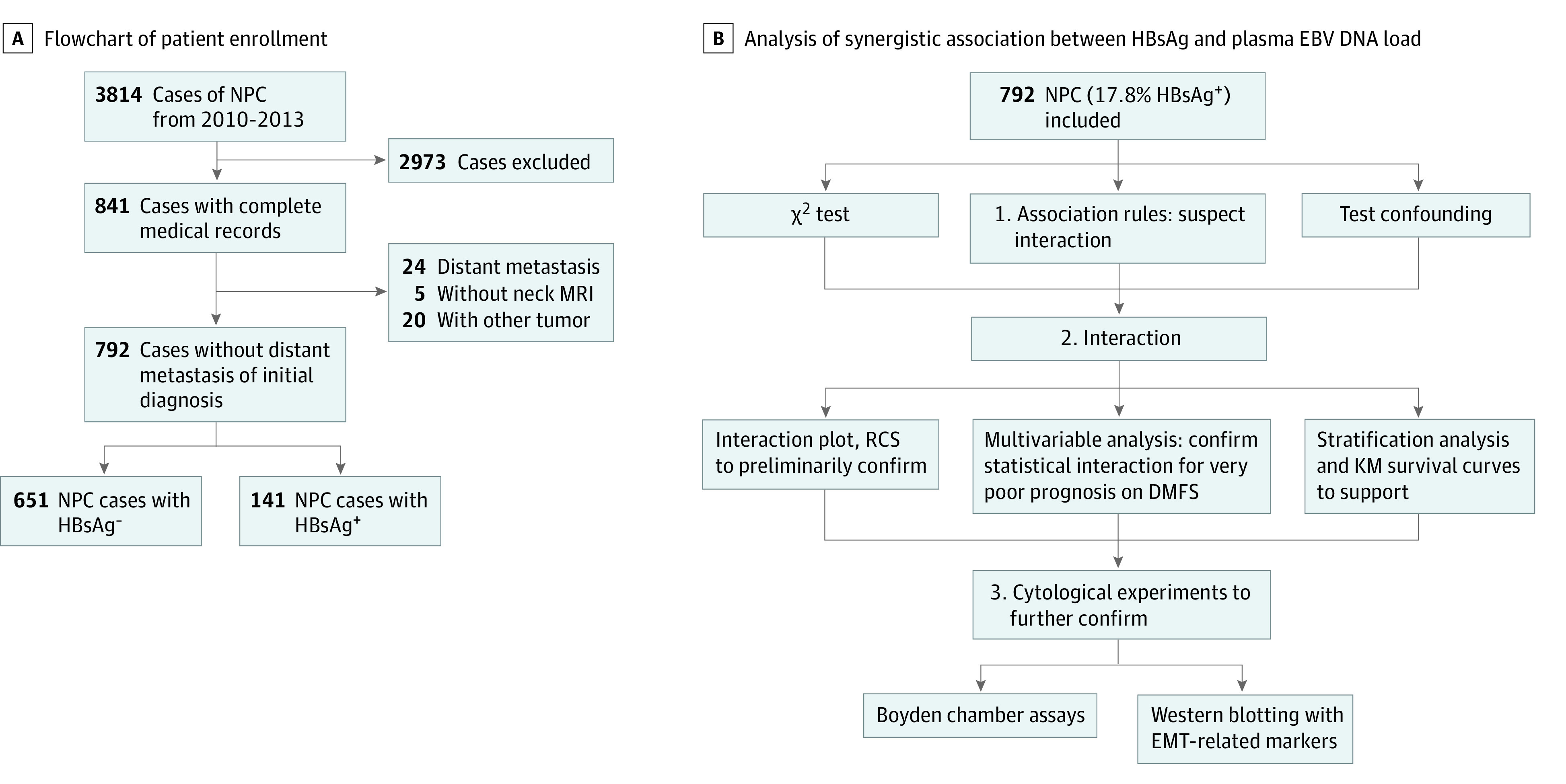
Study Flowchart A, Flowchart of patient enrollment. B, Analysis of synergistic association between hepatitis B surface antigen (HBsAg) and plasma Epstein-Barr (EBV) DNA load. Statistical interaction between plasma EBV DNA load and HBsAg in the distant metastasis–free survival (DMFS) of patients with nasopharyngeal carcinoma (NPC) confirmed in 3 steps. First, we hypothesized the existence of a statistical interaction through “association rules” (eFigure 1 in Supplement 1). HBsAg-positive status and plasma EBV DNA load 1500 copies/mL or greater were closely associated with distant metastasis. Second, we confirmed that a statistical interaction existed between HBsAg positivity and plasma EBV DNA load using interaction plots, restricted cubic spline (RCS) (Figure 2A-B), and multivariable analysis (Figure 2C-D, Table 2). Third, cytological experiments found that HBsAg promoted the invasion and migration of EBV-positive NPC cells via epithelial-mesenchymal transition (EMT) (Figure 3). KM indicates Kaplan-Meier survival analysis; MRI, magnetic resonance imaging.

A total of 792 patients with NPC treated at SYSUCC between January 2010 and January 2013 were enrolled (Figure 1A). The selection criteria were (1) age of 18 to 90 years; (2) confirmed diagnosis of NPC via pathological examination; and (3) complete medical records, including treatment, laboratory tests, and clinical data. The exclusion criteria were (1) presentation with distant metastasis at first clinical visit; (2) presence of other primary tumors besides NPC; and (3) incomplete data on plasma EBV DNA load, HBV detection, and liver function. All participants were restaged according to the 8th edition American Joint Committee on Cancer/Union for International Cancer Control (AJCC/UICC) tumor-node-metastasis (TNM) staging system.

#### Detection of EBV DNA and HBV Markers and Liver Function Assessment

Peripheral blood samples were collected for quantitative detection of plasma EBV DNA and serum HBV markers and liver function assessment. Pretreatment plasma EBV DNA load was measured using real-time fluorescence quantitative polymerase chain reaction before initiating the treatment. Plasma EBV DNA load positivity was defined as 1000 copies/mL or greater, based on a large-scale study.^15^ The hepatitis B–related serological markers tested were HBsAg, hepatitis B surface antibody, hepatitis B e antigen, hepatitis B e antibody, and hepatitis B core antibody. Patients underwent routine hepatitis B detection tests based on enzyme-linked immunosorbent assay at the first clinical visit. Patients with HBsAg positivity (>0.05 IU/mL) were considered HBV-infected. To assess liver function, alanine transaminase (ALT) and aspartate aminotransferase (AST) were evaluated prior to chemotherapy (reference ranges: 9-50 U/L for ALT, 15-40 U/L for AST; to convert to μkat/L, multiply by 0.0167).

#### Treatment

The treatment regimen described in a previously published study^33^ was performed according to the standardized treatment protocols for patients with NPC outlined in the guidelines of our hospital and the National Comprehensive Cancer Network (details provided in eMethods in Supplement 1).

#### Follow-up and Outcomes

Patients were examined each quarter for 2 years and every 6 months during the 5 subsequent years. The primary end point was DMFS, calculated from treatment initiation to distant metastasis. Relevant examination findings were obtained from the hospital’s medical record system. For patients without current follow-up information, telephone interviews were performed.

### Cytological Experiments

#### Cell Lines and Culture Conditions

Human NPC cell lines HK1(EBV-positive), C666-1, HK1(EBV-negative), and 6-10B cells were cultured in complete RPMI-1640 medium (Life Technologies) supplemented with 10% fetal bovine serum (Gibco). HBsAg recombinant proteins (20 ng/mL) were added to the cells, cultured for 48 hours, and harvested for further analysis.

#### Antibodies, Reagents, and Kits

The following reagents were used: human anti-fibronectin, anti-vimentin, and anti-GAPDH antibodies (Abcam), HBsAg (40573-V08E, Sino Biological Inc), DMSO and Transwell chambers (8-mm hole; Corning Inc).

#### NPC Cell Migration and Invasion Assay

Migration of EBV-negative or EBV-positive NPC cells cultured in the presence or absence of HBsAg for 48 hours was assessed. Cells were loaded in Transwell plate chambers for 24 hours, following which the cells in the upper chamber were removed. The cells that penetrated the membrane were stained with crystal violet, and the cells adhering to the membrane surface were enumerated using a microscope (Olympus Corporation).

For cell invasion assays, EBV-negative or EBV-positive NPC cells were cultured with or without HBsAg for 48 hours. The NPC cells were added to matrix gel-coated chambers. After 48 hours, the cells in the upper chamber were removed; those that passed through the membrane were stained with crystal violet and those that adhered to the membrane surface were counted using a microscope (Olympus Corporation).

#### Western Blot Analysis

Proteins were separated by 8% sodium dodecyl sulfate–polyacrylamide gel electrophoresis, transferred to polyvinylidene difluoride membranes (Millipore Corporation), and blocked using 5% fat-free milk. The membranes were further treated overnight with primary antibodies (as described above) and with horseradish peroxide-conjugated secondary antibodies (Santa Cruz) the following day. The proteins were visualized using a chemiluminescence system following x-ray film exposure.

### Statistical Analysis

Distribution differences in clinical characteristics were compared using χ^2^ test or Fisher exact test for categorical variables and *t* test for continuous variables. Data for plasma EBV DNA level were obtained using a previously reported method.^15^ Univariate analysis was performed with the Kaplan-Meier method and compared using the log-rank test. Multivariate analyses were conducted using Cox regression models to calculate hazard ratios (HRs) with 95% CIs and adjusted *P* values. Association rules with the parallel coordinates plot were determined using the “apriori” function from the “arules” package of R software. The interaction plot, created using the “stats” package of R, was used to determine whether HBsAg and plasma EBV DNA level were associated with DMFS. Kaplan-Meier survival curves and restricted cubic spline (RCS) curves were plotted using the “ggplot2” package of R. Maximally selected rank statistics were used to independently determine the plasma EBV DNA level cutoff value. We stratified analyses based on HBsAg status and different plasma EBV DNA levels, ranging from 0.5 to 50 × 1000 copies/mL. The stats, survival, Hmisc, rms, maxstat, ggplot2, apriori, and survminer packages from R (R Foundation for Statistical Computing) were used to conduct all statistical analyses. Detailed summary of the statistical considerations is presented in eMethods in Supplement 1.^34^

For cytological experiments, statistical analysis was performed using GraphPad Prism software, version 9.0 (GraphPad Prism Inc). Data are expressed as mean (SD). Comparisons between 2 groups were performed with 2-tailed unpaired *t* test. *P* < .05 was considered statistically significant.

## Results

### Clinical Characteristics of Patients

Among the 792 patients, 576 (72.7%) were male, with a median (IQR) age of 45 (38-53) years; 141 (17.8%) were HBsAg positive and 651 (82.2%) were HBsAg negative. The median (range) follow-up time was 62.1 (1.4-83.4) months. With a 5-year DMFS of 87.7%, 94 patients developed distant metastasis, and 242 (30.6%) patients were recorded as right-censored data. No significant differences were observed between the HBsAg-positive and HBsAg-negative groups at follow-up.

The distribution of stage, tumor volume, and plasma EBV DNA load were well-balanced between the 2 groups (Table 1). The metastatic rate was marginally higher in the HBsAg-positive groups than in the HBsAg-negative groups (11.5% vs 13.5%; *P* = .56). Although the ALT and AST levels in the HBsAg-positive groups were higher than in the HBsAg-negative groups, they were not associated with DMFS. Moreover, cancer stage and plasma EBV DNA load served as confounding variables in subsequent analyses (eTable 2 and eTable 3 in Supplement 1).^18^

**Table 1.** Sociodemographic and Clinical Characteristics of Participants

Variable	Patients, No. (%)			χ^2^ *P* value^a^	DMFS	
	Total (n = 792)	HBsAg			Survival	*P* value
		Negative (n = 651)	Positive (n = 141)			
Sex						
Female	216 (27.3)	184 (28.3)	32 (22.7)	.20	86.94	.67
Male	576 (72.7)	467 (71.7)	109 (77.3)		88.01	
Age, median (IQR), y	45 (38-53)	45 (38-54)	43 (37-49)	.21	NA	.16
WHO histologic type						
1	5 (0.6)	4 (0.6)	1 (0.7)	.35	80.00	.50
2	41 (5.2)	37 (5.7)	4 (2.8)		81.82	
3	746 (94.2)	610 (93.7)	136 (96.5)		88.08	
Pretreatment plasma EBV DNA level, median (IQR), 1 × 10^3^ copies/mL	1.78 (0-17.2)	1.8 (0-16.5)	1.7 (0-17.3)	.06	NA	.56
Pretreatment plasma EBV DNA level, 1 × 10^3^ copies/mL						
<1	359 (45.3)	294 (45.2)	65 (46.1)	.96	93.57	<.001
<10	185 (23.4)	152 (23.3)	33 (23.4)		83.04	
≥10	248 (31.3)	205 (31.5)	43 (30.5)		82.60	
T classification^b^						
T1	204 (25.8)	173 (26.6)	31 (22.0)	.02	93.95	<.001
T2	97 (12.2)	82 (12.6)	15 (10.6)		88.23	
T3	296 (37.4)	227 (34.9)	69 (48.9)		88.20	
T4	195 (24.6)	169 (26.0)	26 (18.4)		79.88	
N classification^b^						
N0	182 (23.0)	145 (22.3)	37 (26.2)	.24	94.43	<.001
N1	438 (55.3)	367 (56.4)	71 (50.4)		89.18	
N2	113 (14.3)	95 (14.6)	18 (12.8)		77.18	
N3	59 (7.4)	44 (6.8)	15 (10.6)		74.87	
Stage^b^						
I	73 (9.2)	63 (9.7)	10 (7.1)	.06	97.14	<.001
II	175 (22.1)	150 (23.0)	25 (17.7)		93.00	
III	303 (38.3)	235 (36.1)	68 (48.2)		88.52	
IV	241 (30.4)	203 (31.2)	38 (27.0)		79.62	
Treatment						
IMRT	106 (13.4)	89 (13.7)	17 (12.1)	.47	95.10	.03
CCRT	296 (37.4)	237 (36.4)	59 (41.8)		87.68	
IC + CCRT	390 (49.2)	325 (49.9)	65 (46.1)		85.64	
Volume, median (IQR), cm^3^	30.0 (17.5-49.9)	30.2 (17.3-49.8)	29.9 (18.1-50.5)	.57	NA	<.001
HBsAg						
Negative	651 (82.2)	651 (100)	0	<.001	88.13	.44
Positive	141 (17.8)	0	141 (100)		85.69	
HBsAb						
Negative	352 (44.4)	211 (32.4)	141 (100)	<.001	89.07	.34
Positive	440 (55.6)	440 (67.6)	0		86.63	
HBeAg						
Negative	775 (97.9)	651 (100)	124 (87.9)	<.001	87.60	.55
Positive	17 (2.1)	0	17 (12.1)		93.33	
HBeAb						
Negative	572 (72.2)	540 (82.9)	32 (22.7)	<.001	87.89	.81
Positive	220 (27.8)	111 (17.1)	109 (77.3)		87.25	
HBcAb						
Negative	510 (64.4)	499 (76.7)	11 (7.8)	<.001	87.95	.76
Positive	282 (35.6)	152 (23.3)	130 (92.2)		87.30	
ALT, U/L						
<50	721 (91.0)	604 (92.8)	117 (83.0)	<.001	87.52	.66
≥50	71 (9.0)	47 (7.2)	24 (17.0)		89.58	
AST, U/L						
<40	756 (95.5)	631 (96.9)	125 (88.7)	<.001	87.40	.25
≥40	36 (4.5)	20 (3.1)	16 (11.3)		94.12	

Abbreviations: ALT, alanine aminotransferase; AST, aspartate transaminase; CCRT, concurrent chemoradiotherapy; DMFS, distant metastasis–free survival; EBV, Epstein-Barr virus; HBcAb, hepatitis B core antibody; HBeAb, hepatitis B e antibody; HBeAg, hepatitis B e antigen; HBsAb, hepatitis B surface antibody; HBsAg, hepatitis B surface antigen; IC, induction chemotherapy; IMRT, intensity-modulated radiotherapy; NA, not applicable; WHO, World Health Organization.

SI conversion factor: To convert ALT and AST to μkat/L, multiply by 0.0167.

^a^ *P* values were calculated using Fisher exact test or χ^2^ test for categorical variables and *t* test for continuous variables.

^b^ According to the 8th edition of the American Joint Committee on Cancer/Union for International Cancer Control staging system.

### Interaction Between HBsAg and Plasma EBV DNA Load on DMFS in Patients With NPC

In total, 1049 rules were identified based on the rules of association, and the top 20 rules were presented on a parallel coordinates plot (eFigure 1A and eTable 1 in Supplement 1). Given that the T/N stage is associated with DMFS and that normal AST and ALT levels are not associated with DMFS, EBV-related and HBV-related proteins were used to reassess and plot the top 10 association rules (eFigure 1B and eTable 1 in Supplement 1). HBsAg-positive status with plasma EBV DNA levels 1500 copies/mL or greater exhibited the strongest association with distant metastasis.

**Figure 2.**
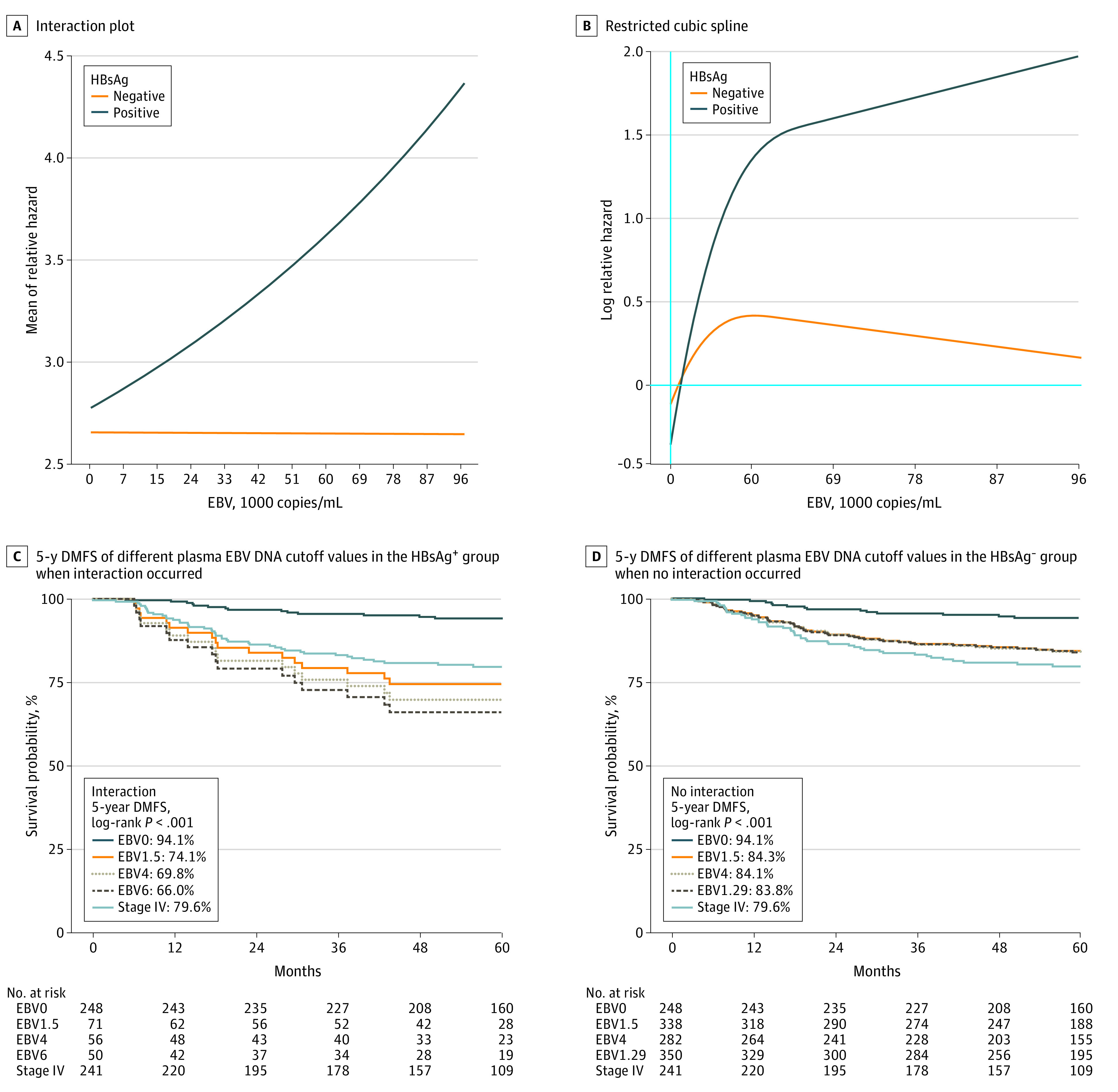
Interaction, Restricted Cubic Spline (RCS) Curve, and Survival Analysis A, Interaction plot: nonparallel curves between the hepatitis B surface antigen (HBsAg)-positive and HBsAg-negative groups indicate the statistical interaction between HBsAg positivity and the plasma Epstein-Barr virus (EBV) DNA load. B, RCS: risk (log relative hazard) of distant metastasis in the HBsAg-positive group increases along with the increase of plasma EBV DNA load. C, The 5-year distant metastasis–free survival (DMFS) of different plasma EBV DNA cutoff values in the HBsAg-positive group when interaction occurred. D, The 5-year DMFS of different plasma EBV DNA cutoff values in HBsAg-negative group when no interaction occurred. EBV0, Stage IV referred to patients in the total cohort. Plasma EBV DNA level of 0 was set as a reference value for survival analysis; the cutoff value of 1.5 × 1000 copies/mL represented the starting point of a significant interaction; the cutoff value of 4 × 1000 copies/mL (between 1.5 × 1000 copies/mL to 6 × 1000 copies/mL) illustrated the tendency of interaction along the changes in plasma EBV DNA load (Table 2); the cutoff value of 6 × 1000 copies/mL represented the most obvious interaction (Table 2) and the optimal value for predicting DMFS in the HBsAg-positive group; the cutoff value of 1.29 × 1000 copies/mL represented the optimal value for predicting DMFS in the HBsAg-negative group (eFigure 2 in Supplement 1). Multivariable Cox regression analysis results for Figure 2C-D are presented in eTable 3 in Supplement 1. EBV0 indicates plasma EBV DNA level = 0; EBV1.5, plasma EBV DNA level ≥1.5 × 1000 copies/mL, etc.

The interaction plot highlights a statistical interaction between HBsAg-positive status and plasma EBV DNA load (Figure 2A). The increasing trend of distant metastasis in HBsAg-positive patients was visualized using RCS, along with increase in the plasma EBV DNA load (Figure 2B). In the stratification analysis (Table 2, Step 1), when values greater than the EBV DNA cutoff value were compared with those lower, the HBsAg-positive group (HR range, 1.69-9.90) consistently exhibited a higher risk than the HBsAg-negative group (HR range, 0.95-1.88), which was confirmed using RCS (Figure 2B; eTable 4 in Supplement 1). The interaction between HBsAg and plasma EBV DNA levels was observed when plasma EBV DNA cutoff values were 1.5 × 1000 copies/mL or greater (*P* < .05; supporting data in Table 2, Step 2).

**Table 2.** Interaction Confirmed by Multivariable Cox Regression Analysis

EBV DNA cutoff, ×1000 copies/mL	Step 1: stratification analysis^a^						Step 2: interaction	
	HBsAg negative (n = 651)			HBsAg positive (n = 141)				
	No.	Multivariate AJCC/UICC-adjusted		No.	Multivariate AJCC/UICC-adjusted			
		HR (95% CI)	*P* value		HR (95% CI)	*P* value	HR (95% CI)	*P* value
0.5	398	1.75 (1.01-3.02)	.05	83	5.45 (1.25-23.76)	.02	3.39 (0.71-16.11)	.13
1	357	1.88 (1.12-3.16)	.02	76	6.90 (1.58-30.10)	.01	3.97 (0.84-18.7)	.08
1.5^b^	338	1.75 (1.06-2.89)	.03	71	8.49 (1.95-36.89)	.004	5.17 (1.10-24.21)	.04
2	321	1.72 (1.05-2.81)	.03	67	9.51 (2.19-41.29)	.003	5.87 (1.26-27.46)	.02
3	297	1.66 (1.03-2.69)	.04	58	7.86 (2.28-27.06)	.001	5.01 (1.34-8.74)	.02
4	282	1.54 (0.96-2.48)	.07	56	8.57 (2.49-29.49)	.001	5.83 (1.56-21.78)	.009
5	263	1.26 (0.79-2.01)	.32	52	9.60 (2.78-33.11)	<.001	7.98 (2.14-29.71)	.002
6^c^	244	1.13 (0.70-1.80)	.61	50	9.90 (2.85-34.33)	<.001	9.16 (2.46-34.14)	.001
7	226	0.95 (0.59-1.54)	.86	46	5.51 (1.95-15.55)	.001	6.06 (1.97-18.63)	.002
8	217	1.03 (0.64-1.66)	.90	45	5.75 (2.04-16.21)	.001	5.86 (1.91-18.03)	.002
9	213	1.07 (0.66-1.72)	.77	43	6.16 (2.17-17.47)	.001	6.02 (1.96-18.53)	.002
10	205	1.06 (0.65-1.71)	.81	43	6.16 (2.17-17.47)	.001	6.09 (1.98-18.77)	.002
20	154	1.01 (0.60-1.70)	.96	31	3.73 (1.48-9.36)	.005	3.93 (1.39-11.06)	.01
30	124	0.99 (0.56-1.74)	.99	28	4.49 (1.79-11.26)	.001	4.79 (1.67-13.79)	.004
40	104	1.04 (0.58-1.88)	.88	21	2.29 (0.86-6.07)	.10	2.39 (0.77-7.38)	.13
50	88	1.18 (0.64-2.16)	.59	16	1.69 (0.55-5.14)	.35	1.58 (0.45-5.54)	.48

Abbreviations: AJCC/UICC, American Joint Committee on Cancer/Union for International Cancer Control; EBV, Epstein-Barr virus; HBsAg, hepatitis B surface antigen; HR, hazard ratio; RCS, restricted cubic spline.

^a^ Step 1: in stratification analysis, when the higher plasma EBV DNA cutoff value was compared with the lower one, the HBsAg-positive group (HR, 1.69-9.90) always presented a greater risk than the HBsAg-negative group (HR, 0.95-1.88), which could also be visualized using RCS (Figure 2). This is also presented in eTable 4 in Supplement 1 (setting plasma EBV DNA level = 0 as the same reference group in both HBsAg-positive and HBsAg-negative groups made the HR in the 2 groups comparable). As an explanation for this finding, the significant interaction confirmed that using multivariable Cox regression could be a major reason for the observation (when plasma EBV DNA cutoff value ranged from 1.5 × 1000 copies/mL to 30 × 1000 copies/mL, *P* < .05).

^b^ 1.5 × 1000 copies/mL was the start point of significant interaction in HBsAg-positive group. EBV DNA cutoff = 1.5 indicates the comparison between plasma EBV DNA levels 1500 copies/mL or greater vs plasma EBV DNA levels less than 1500 copies/mL, and the number of patients indicates the number of patients with plasma EBV DNA levels 1500 copies/mL or greater; the lower one was set as the reference to calculate the HR and *P* values using multivariable Cox regression with stage as a confounding variable. The details of the multivariable Cox regression analysis are provided in eTables 2 and 3 in Supplement 1.

^c^ 6 × 1000 copies/mL was the best cutoff value of EBV DNA in HBsAg-positive group (as presented in eFigure 2B in Supplement 1). The highest metastasis risk (HR) in the HBsAg-positive group was 9.90 (95% CI, 2.85-34.33), when comparing patients with plasma EBV DNA levels 6000 copies/mL or greater and those with plasma EBV DNA levels less than 6000 copies/mL.

### Risk of Distant Metastasis Associated With Interaction Between HBsAg and EBV

By including all patients with plasma EBV DNA levels of 0 in the same reference group, the HRs for metastasis in the HBsAg-positive and HBsAg-negative groups were comparable (eTable 4 in Supplement 1). A significantly higher risk was observed in the HBsAg-positive group (HR range, 2.85-6.02) than that in the HBsAg-negative group (HR range, 1.82-2.29) at plasma EBV DNA levels of 2 to 30 × 1000 copies/mL or greater. Compared with the HR for distant metastasis at plasma EBV DNA level of 0, the HR in the HBsAg-positive group increased gradually from 2.85 to 6.02 when the plasma EBV DNA level increased from 0 to 30 × 1000 copies/mL, which was not observed in the HBsAg-negative group. Similarly, in the Kaplan-Meier survival analysis, DMFS decreased gradually with an increase in plasma EBV DNA level in the HBsAg-positive group (Figure 2C). When no interaction was observed, the survival curves of different cutoff values (>0) overlapped, which was worse than those with plasma EBV DNA level of 0 but better than patients with stage IV disease (Figure 2D). The most appropriate plasma EBV DNA cutoff values for predicting DMFS in the HBsAg-positive and HBsAg-negative groups were 6 × 1000 and 1.29 × 1000 copies/mL, respectively (eFigure 2 in Supplement 1). Notably, the risk in the HBsAg-positive group was twice than that in patients with stage IV disease (final-stage NPC) at plasma EBV DNA levels 6 × 1000 copies/mL or greater (5-year DMFS 66% vs 79.6%; *P* = .04; HR, 2.01; 95% CI, 1.13-3.56; *P* = .02; Figure 2C).

### Association of HBsAg With Migration and Invasion of EBV-Positive NPC Cells Through EMT

**Figure 3.**
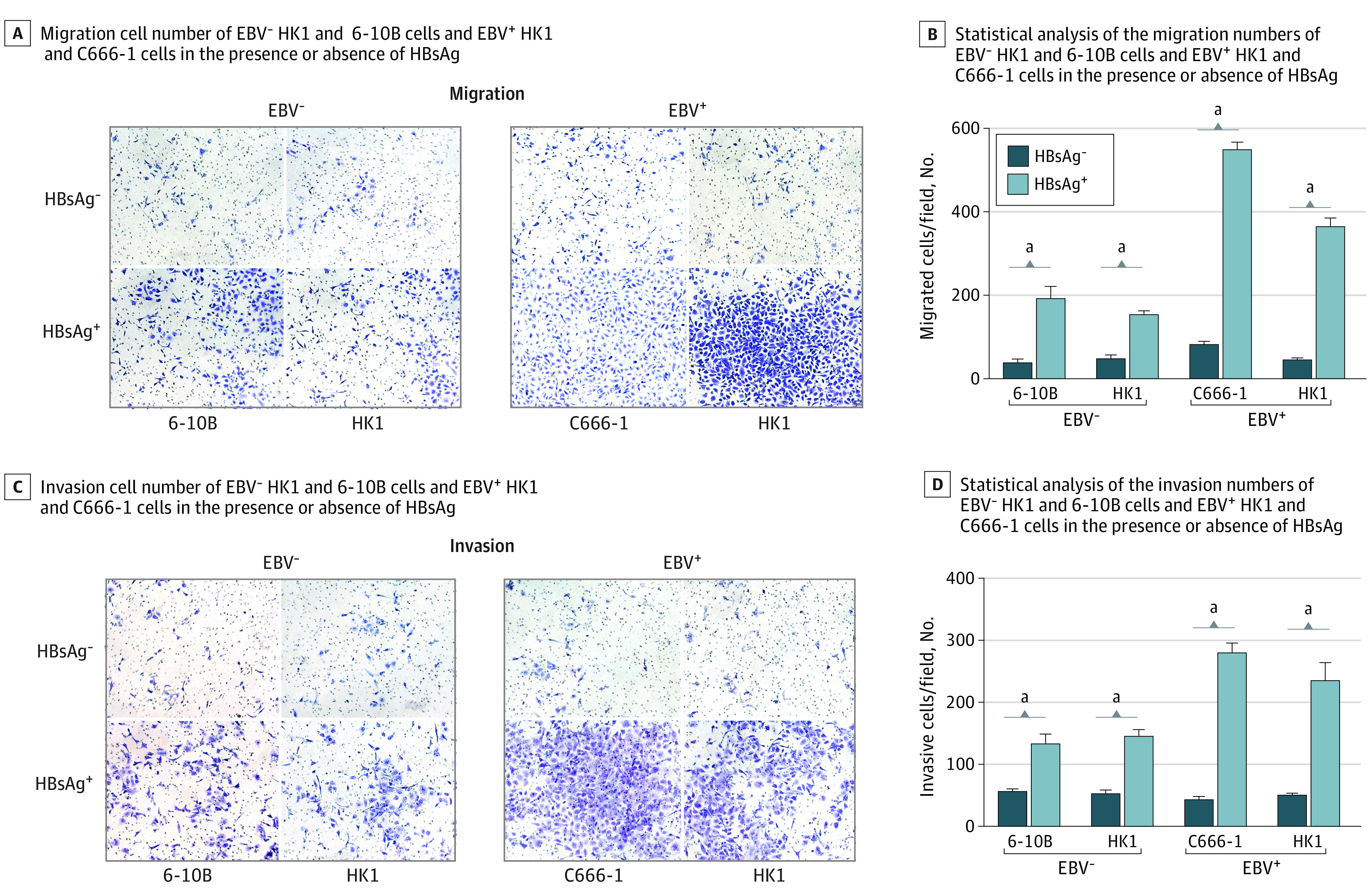
Migration and Invasion of EBV-Positive Nasopharyngeal Carcinoma Cells Promoted by HBsAg Through Epithelial-Mesenchymal Transition A, Epstein-Barr virus (EBV)-negative HK1 and 6-10B cells and EBV-positive HK1 and C666-1 cells were treated with hepatitis B surface antigen (HBsAg) or incubated in the medium for 48 hours. The migration cell number of EBV-negative HK1 and 6-10B cells and EBV-positive HK1 and C666-1 cells in the presence or absence of HBsAg was determined using the migration assays in Transwell chambers. The migrating cells are indicated by the average number of cells per field of view from at least 3 different independent experiments. B, Statistical analysis of the migration numbers of EBV-negative HK1 and 6-10B cells and EBV-positive HK1 and C666-1 cells in the presence or absence of HBsAg. C, The invasion cell number of EBV-negative HK1 and 6-10B cells and EBV-positive HK1 and C666-1 cells in the presence or absence of HBsAg was determined using the invasion assays in Matrigel-coated Transwell chambers. The invasive cells are indicated by the average number of cells per field of view from at least 3 different independent experiments. D, Statistical analysis of the invasion numbers of EBV-negative HK1 and 6-10B cells and EBV-positive HK1 and C666-1 cells in the presence or absence of HBsAg. All tests were repeated 3 times. Statistical significance was determined using the *t* test. Data presented are means; error bars indicate SD. ^a^*P* < .001.

Based on our findings that HBV infection is associated with high metastasis in patients with NPC with high EBV load, we used the Boyden chamber assay to investigate whether HBsAg is associated with the aggressive migration and invasion of EBV-positive NPC cells. HBsAg significantly promoted cell migration and invasion in NPC cells, particularly within EBV-positive cells (Figure 3). Furthermore, the abundance of fibronectin and vimentin—mesenchymal markers—were significantly upregulated in EBV-positive NPC cells, not EBV-negative NPC cells (eFigure 3 in Supplement 1). Hence, HBsAg promoted the capacity of migration and invasion of EBV-positive NPC cells via EMT. These results were supported by cytological analyses.

## Discussion

This cohort study explores the potential mechanism underlying metastasis in patients with EBV-associated NPC with HBsAg-positive status by combining clinical findings with cytological evidence. HBsAg promoted the migration and invasion of EBV-positive NPC cells through EMT-related pathways, which might account for significant worsening of the DMFS in response to interactions between HBsAg-positive status and different plasma EBV DNA loads in patients with NPC. In fact, the HR for distant metastasis increased with increasing plasma EBV DNA load only in the HBsAg-positive group. Moreover, metastatic risk was consistently higher in the HBsAg-positive group than the HBsAg-negative group (highest difference, up to 9.90-fold).

Previous studies have demonstrated that the HR for distant metastasis varies with different cutoff values of plasma EBV DNA level,^16,18,35,36,37,38,39^ indicating that plasma EBV DNA load is associated with the risk for distant metastasis. These findings support the increasing HR trend in the HBsAg-positive group. Additionally, the HR for DMFS differed in various studies, even with the cutoff value set at 4000 copies/mL,^18,37,38,39^ suggesting that different confounding factors influence the distant metastasis HR when the plasma EBV DNA load is constant. Meanwhile, in the current study, the DMFS HR increased substantially in the presence of HBsAg when plasma EBV DNA cutoff value was constant. Evidence from a former study^22^ supports our finding that the coexistence of HBsAg and plasma EBV DNA is associated with worse DMFS in patients with NPC. However, the former study focused on HR when the plasma EBV DNA load was set at 1500 copies/mL, without considering trends at other plasma EBV DNA cutoff values. We also show that the distant metastasis HR increases with increasing plasma EBV DNA load in HBsAg-positive patients with NPC. Through multivariate and Kaplan-Meier survival analyses, we calculated the HR for DMFS in HBsAg-positive patients with NPC at different EBV DNA cutoff values and further visualized the interaction between HBsAg and plasma EBV DNA load statistically. Hence, plasma EBV DNA load and HBsAg status should be considered when stratifying the risk of distant metastasis in patients with NPC. Indeed, incorporation of plasma EBV DNA level into a prognostic nomogram improves the predictive power of the conventional nomogram based on the AJCC/UICC TNM classification.^15^ Therefore, knowledge regarding the statistical interaction between HBsAg and plasma EBV DNA load in the prognostic nomogram may help develop better disease management strategies. In the terms of accurate medication, clinical trials such as immunotherapy trials might be an option for patients with NPC with HBsAg positivity, particularly for patients with advanced-stage disease or with high plasma EBV DNA load.

Why does the risk of distant metastasis increase upon the interaction between HBsAg and plasma EBV DNA load in NPC? Our in vitro experiments suggest that HBsAg promotes the migration and invasion of EBV-positive cells through EMT-related pathways. EBV and HBV proteins influence EMT in different cell types.^28,29,30,31,32^ We hypothesized that HBsAg promotes NPC metastasis via an EBV-related metastatic mechanism. This hypothesis was supported by Western blot analysis when HBsAg treatment increased the protein levels of fibronectin and vimentin in EBV-positive NPC cells but not in EBV-negative NPC cells. Hence, HBsAg was shown to promote the metastasis of EBV-positive NPC cells via EMT. That is, HBV infection promotes inflammatory cell infiltration and immune function impairment, making patients with NPC more susceptible to distant metastasis.^20,21,22,23^ Hence, identification of this EMT-related mechanism may improve the individualized treatment of HBsAg-positive patients with NPC. However, further studies to elucidate the immunological mechanism and EMT-related pathways are warranted.

The percentage of HBsAg-positive patients with NPC in the current study was 17.8%, which was within the current reported infection rates (10.3%-24.8%)^19,31^ and higher than the HBV incidence rate in endemic areas (average, 10%-12%).^40^ The finding that HBV infection is associated with higher NPC risk^41^ may help explain this phenomenon. There was no significant difference in stage, tumor volume, or EBV status between patients in the HBsAg-positive and HBsAg-negative groups, indicating that HBsAg status was not significantly associated with tumor burden. Therefore, synergistic association between HBsAg and plasma EBV DNA load is associated with significantly worse DMFS among patients with NPC with HBsAg positivity.

### Limitations

Owing to its retrospective nature, the small sample size, and undocumented medical records used in this study, including HBV DNA load, cannot be ignored. Since our hospital specializes in treating patients with cancer, the HBV DNA load was not routinely measured, and patients were more likely to receive hepatitis therapy in other hospitals. Therefore, a large-scale multicenter study is warranted. Additionally, a better understanding of the molecular mechanism underlying EMT induction by HBsAg in EBV-positive NPC cells would aid precision medication and improve prognosis. Hence, investigation of the potential mechanistic effect of HBsAg in vivo is needed to strengthen our findings. Immunological experiments on metastasis are necessary to validate previous hypotheses. Based on specified risk stratification, it is necessary to explore follow-up principles and individualized treatments for patients with NPC with different EBV DNA loads and HBsAg statuses.

## Conclusions

In this cohort study, HBsAg positivity and a higher plasma EBV DNA level (1.5-30 × 1000 copies/mL) were associated with a significantly increased risk for metastasis in patients with NPC, indicating a synergistic association between HBsAg and plasma EBV DNA load. Cytological experiments found that HBsAg promoted the invasion and migration of EBV-positive NPC cells through inducing EMT. Hence, patients with NPC with HBsAg positivity should be monitored for distant metastasis, particularly when the plasma EBV DNA load is 6 × 1000 copies/mL or greater. Moreover, this synergistic association should be considered for accurate risk stratification of patients with NPC. Our finding that HBsAg may trigger the EMT pathway in EBV-positive NPC cells provides a novel perspective on the mechanisms underlying distant metastasis.

## Notes

Supplement 1.
eMethods.
**eFigure 1.** Close association between HBsAg(+), plasma EBV DNA load, and distant metastasis **eFigure 2.** Best plasma EBV DNA load cutoff point **eFigure 3.** HBsAg induced the epithelial-mesenchymal transition of EBV(+) NPC cells **eTable 1.** Original association rules **eTable 2.** Test of confounding variables and samples of interaction effect **eTable 3.** Stratification analysis based on HBsAg(+/-) status or different plasma EBV DNA cutoff values **eTable 4.** Quantification of risk between the HBsAg(+) and HBsAg(-) groups at different plasma EBV DNA loads

Supplement 2.
Data Sharing Statement
