# Supplementary material for: Synergistic Association of Hepatitis B Surface Antigen and Plasma Epstein-Barr Virus DNA Load on Distant Metastasis in Patients With Nasopharyngeal Carcinoma
Source: JAMA Netw Open. 2023 Feb 9;6(2):e2253832. doi: 10.1001/jamanetworkopen.2022.53832 (PMC9912125; doi:10.1001/jamanetworkopen.2022.53832)
Supplement: Supplement 1. — eMethods. eFigure 1. Close association between HBsAg(+), plasma EBV DNA load, and distant metastasis eFigure 2. Best plasma EBV DNA load cutoff point eFigure 3. HBsAg induced the epithelial-mesenchymal transition of EBV(+) NPC cells eTable 1. Original association rules eTable 2. Test of confounding variables and samples of interaction effect eTable 3. Stratification analysis based on HBsAg(+/-) status or different plasma EBV DNA cutoff values eTable 4. Quantification of risk between the HBsAg(+) and HBsAg(-) groups at different plasma EBV DNA loads [file jamanetwopen-e2253832-s001.pdf]

Supplemental Online Content

Li H, Cao D, Li S, et al. Synergistic association of hepatitis B surface antigen and plasma Epstein-Barr virus DNA load on distant metastasis in patients with nasopharyngeal carcinoma. *JAMA Netw Open.* 2023;6(2):e2253832. doi:10.1001/jamanetworkopen.2022.53832

eMethods.

eFigure 1. Close association between HBsAg(+), plasma EBV DNA load, and distant metastasis

eFigure 2. Best plasma EBV DNA load cutoff point

eFigure 3. HBsAg induced the epithelial-mesenchymal transition of EBV(+) NPC cells

eTable 1. Original association rules

eTable 2. Test of confounding variables and samples of interaction effect

eTable 3. Stratification analysis based on HBsAg(+/-) status or different plasma EBV DNA cutoff values

eTable 4. Quantification of risk between the HBsAg(+) and HBsAg(-) groups at different plasma EBV DNA loads

This supplemental material has been provided by the authors to give readers additional information about their work.

© 2023 Li H et al. *JAMA Network Open.*

eMethods

Treatment

All patients underwent intensity-modulated radiation therapy (IMRT) during the radiotherapy course. The planning target volume to primary gross tumor volume and nodal gross tumor volume were 68 Gy and 60–64 Gy, respectively; 60 Gy was the prescribed dose for the clinical target volume with high risk, whereas 54 Gy was the prescribed dose for clinical target volume with low risk and neck nodal regions. All the patients received IMRT with one fraction per day for 6–7 weeks (5 days per week). A total of 106 patients (13.4%) received IMRT alone, 296 patients (37.4%) received cisplatin-based concurrent chemotherapy (30–40 mg/m^2 cisplatin per week or 80–100 mg/m^2 cisplatin for two to three cycles over 3 weeks), and 390 patients (49.2%) received additional IC plus concurrent chemoradiotherapy (80 mg/m^2 cisplatin plus 1000 mg/m^2 5-fluorouracil, 75 mg/m^2 cisplatin plus 75 mg/m^2 docetaxel, or 60 mg/m^2 cisplatin plus 5-600 mg/m^2 fluorouracil plus 60 mg/m^2 docetaxel for two to three cycles over 3 weeks). Salvage therapy, such as secondary radiation, surgery, and chemotherapy, was performed if patients relapsed or exhibited persistent symptoms.

Statistical analysis

The formula used for the statistical interaction effect in Cox regression was $Z = aX + bY + c(XY)$. $aX + bY$, the variable on the left-hand side of the formula was known. $c(XY)$ represented the interaction effect. When the interaction effect occurred, the *P* value for the coefficient of (XY) was expected to be significant.[1]

Association rules with the parallel coordinates plot were determined using the “apriori” function from the “arules” package of R software (version 3.2.5; R Foundation for Statistical Computing, http://www.r-project.org) and used to explore the relationship between different variables (including plasma EBV DNA levels, hepatitis B detection-related antigens and antibodies, and ALT and AST levels) and distant metastasis. The interaction plot created using the “stats” package of R was used to determine whether HBsAg and plasma EBV DNA level exhibited an interaction effect on DMFS.

Association rules

Association rules are widely used in data mining and knowledge discovery. In this study, association rules use the Apriori algorithm to mine frequent itemsets by employing a level-wise search.[2] Commonly, two aspects form the association rule: the left hand side items (LHS) which occur first by one, or a combination of several items. In the current study, the LHS items included plasma EBV DNA levels, hepatitis B detection-related antigens and antibodies, and ALT and AST levels. The right hand side items (RHS, or Consequence) occur later, and were set as distant metastasis in our study. The occurrence of certain events (LHS) would frequently cause the occurrence of others (RHS).

Association rules contain many indicators, including “support”,”lift”,”confident”, etc. However, in this study, identification of the top N rules with relative higher correlation between LHS (plasma EBV DNA levels, hepatitis B detection-related antigens, etc.) and RHS (distant metastasis) was the primary target. Hence, we selected “lift” as an indicator in this study. When “lift” is > 1, the higher the value is, the stronger the positive correlation is between the LHS and RHS; when “lift” is < 1, the lower the value is, the stronger the negative correlation is between the LHS and RHS; when “lift” = 1, there is no correlation between the LHS and RHS.

A parallel coordinate plot helps to visualize the association rules result. Within these plots, the Y-axis represents the variables, while the X-axis presents the number of variables included in the rule. For example, the largest N = 6 indicates that the most complex rules had six variables involved. The last or the most important “position” was RHS (Consequence) which is explained above. Each rule corresponds to a polyline. The line in the plot represents multiple polylines overlapping. The thickness of the curves represents the occurrence frequency of each association rule; the thicker the curve, the more frequent the association rule occurs, indicating a higher correlation with RHS. RHS occur after LHS, however, items in LHS can occur simultaneously or successively without a certain correlation, at least in our study.

References

1. James G, Witten D, Hastie T, Tibshirani R. Statistical Learning. In: Casella G, Fienberg S, Olkin I, eds. *An Introduction to Statistical Learning.* Springer, 2013:15–58. doi:10.1007/978-1-4614-7138-7.
2. Borgelt C. Frequent item set mining. *Wiley Interdiscip Rev: Data Min Knowl Discov*. 2012;2(6):437–456. doi:10.1002/widm.1074

© 2023 Li H et al. *JAMA Network Open.*

eFigures

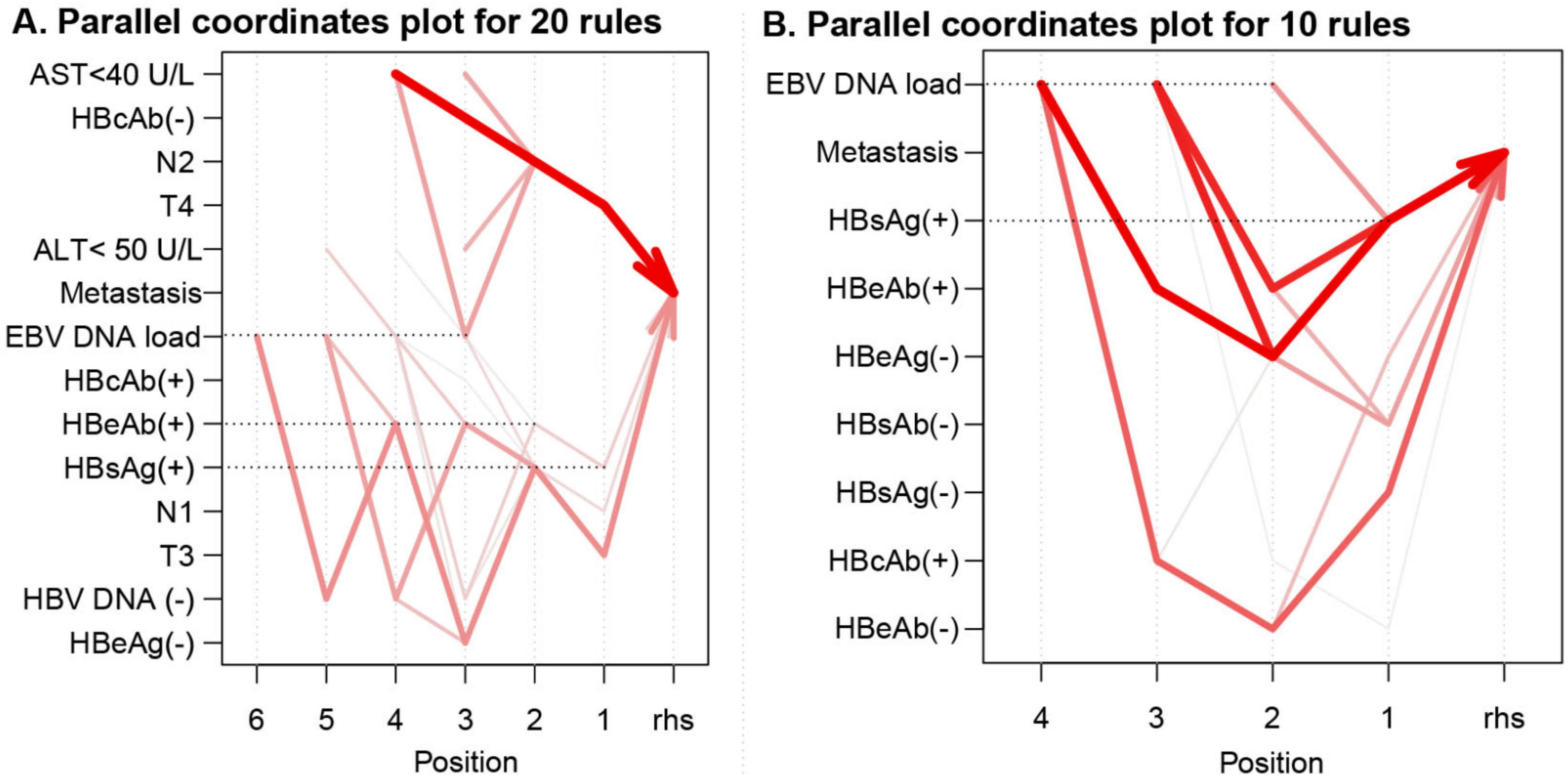

eFigure 1. Close association between HBsAg(+), plasma EBV DNA load, and distant metastasis

A, Top 20 multivariable association rules associated with DMFS are plotted because T/N classification was known to be associated with DMFS; however, the new rules seemed to indicate the involvement of EBV- and HBV-related proteins. AST and ALT levels did not add any information. B, Exclusion of T/N stage and AST and ALT levels and inclusion of EBV- and HBV-related proteins for reanalysis of association rules and plotting of the top 10 rules. Most rules indicated the involvement of EBV and HBsAg. This suggests that the association between HBsAg(+) and EBV(+) statuses affects DMFS. The original rules were presented in **eTable1 in Supplement.**

Note: The thickness of the curves represents the occurrence frequency of each association rule. X-axis: the number of the position indicates the number of variables included in the rule. Rhs was set as distant metastasis, which is the consequence of this study. For variables in Y-axis, EBV DNA load is defined as Plasma EBV DNA load $\geq 1.5 \times 1000$ copies/mL; HBV DNA (-) is defined as plasma HBV DNA load < 1000 copies/mL or missing data.

Abbreviations: DMFS, distant metastasis-free survival; EBV, Epstein–Barr virus, HBV, hepatitis B virus; ALT, alanine transaminase; AST, aspartate aminotransferase; HBsAg, hepatitis B surface antigen; rhs, right hand side item.

© 2023 Li H et al. *JAMA Network Open.*

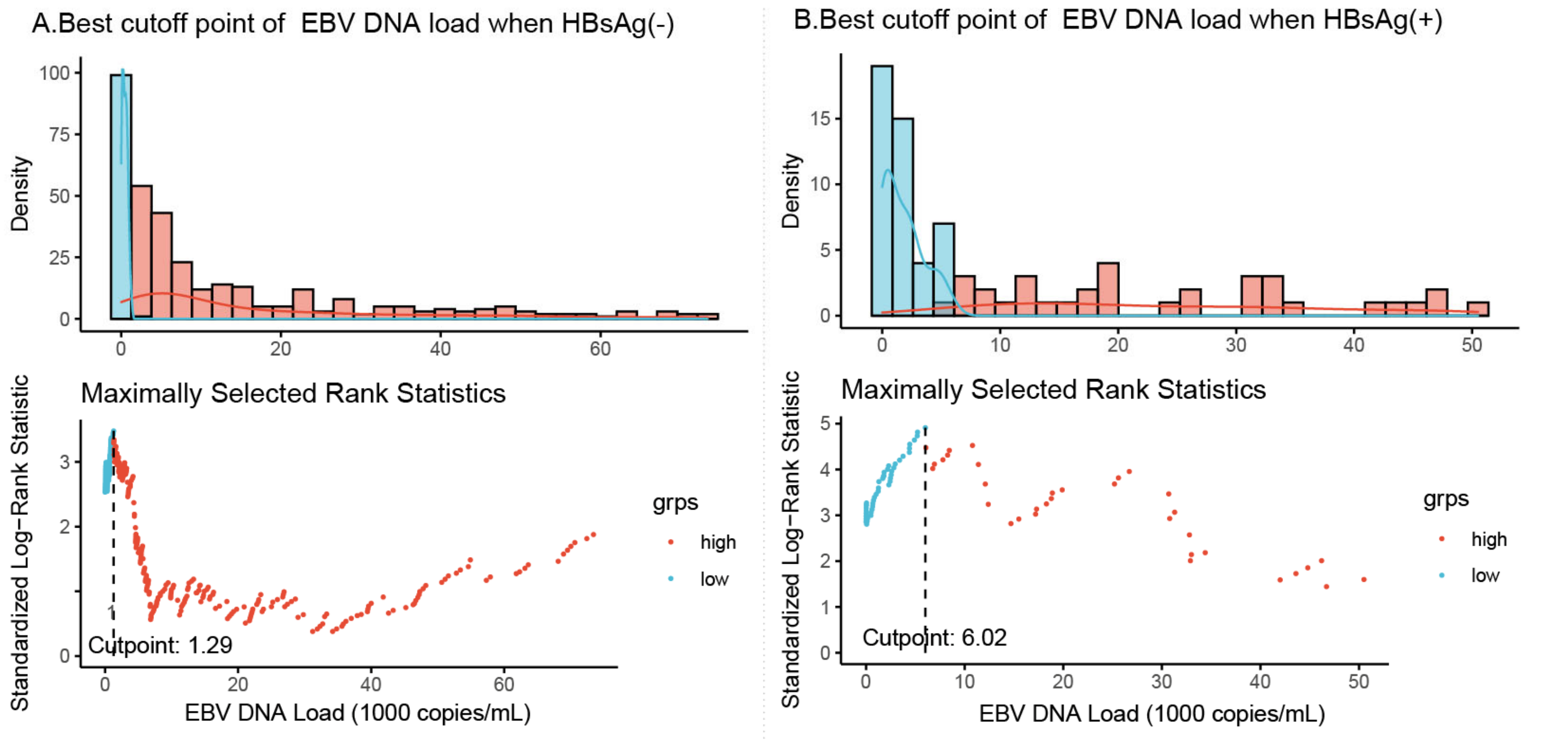

eFigure 2. Best plasma EBV DNA load cutoff point

Note 1: As observed in the maximally selected rank statistics for the determination of the best cutoff value that maximized the group differences, different cutoff values (1.29 × 1000 copies/mL and 6.02 × 1000 copies/mL) were identified based on the different HBsAg(+/-) statuses.

Note 2: In the HBsAg(+) group, the cutoff value was 6.02 × 1000 copies/mL, which was considerably close to 6 × 1000 copies/mL; accordingly, in all subsequent statistical analyses, we used 6 × 1000 copies/mL as the cutoff value.

Abbreviations: HBsAg, hepatitis B surface antigen; EBV, Epstein–Barr virus; +, positive; -, negative.

© 2023 Li H et al. *JAMA Network Open.*

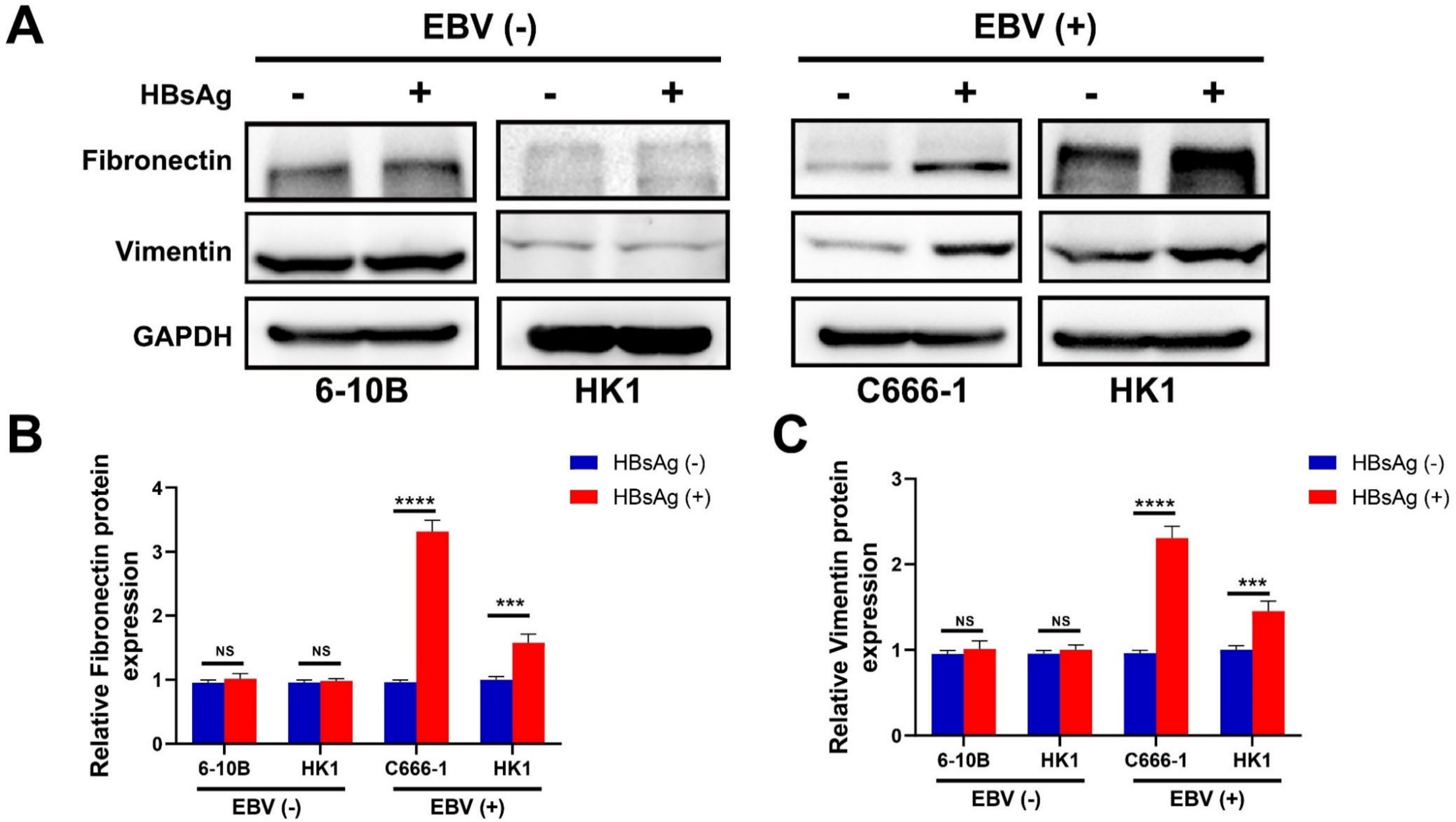

eFigure 3. HBsAg induced the epithelial-mesenchymal transition of EBV(+) NPC cells.

A. Immunoblotting for measuring the key EMT-related proteins including fibronectin and vimentin in the EBV(-) HK1 and 6-10B cells and EBV(+) HK1 and C666-1 cells in the presence or absence of HBsAg. GAPDH was used as the control. B,C. Relative intensity of fibronectin and vimentin in the EBV(-) HK1 and 6-10B cells and EBV(+) HK1 and C666-1 cells in the presence or absence of HBsAg. All tests were repeated three times.

Note: Statistical significance was determined using the Student's t-test. Error bars indicate mean ± SD.*P < .05, **P < .01, ***P < .001, and ****P < .0001.

Abbreviations: HBsAg, hepatitis B surface antigen; EBV, Epstein–Barr virus; +, positive; -, negative; EMT, epithelial-mesenchymal transition; SD, standard deviation; NPC, nasopharyngeal carcinoma.

© 2023 Li H et al. *JAMA Network Open.*

eTables

eTable 1. Original association rules

No.	lhs(rules)	support	confidence	coverage	lift	count
**Top 20 rules associated with DMFS**						
[1]	{Tstage=4,Nstage=2,HBcAb=0,AST_pre=0}	0.014	0.458	0.030	3.862	11
[2]	{Tstage=4,Nstage=2,HBcAb=0}	0.014	0.440	0.032	3.707	11
[3]	{Tstage=3,HBsAg=1,HBeAg=0,HBeAb=1,HBV_DNA_active=0,EBV=1}	0.014	0.393	0.035	3.310	11
[4]	{Tstage=4,Nstage=2, EBV=1,AST_pre=0}	0.014	0.379	0.037	3.196	11
[5]	{Tstage=3,HBsAg=1,HBeAb=1,HBV_DNA_active=0, EBV=1}	0.014	0.379	0.037	3.196	11
[6]	{Tstage=4,Nstage=2,AST_pre=0}	0.015	0.375	0.040	3.160	12
[7]	{Tstage=4,Nstage=2, EBV=1}	0.014	0.367	0.038	3.089	11
[8]	{Tstage=4,Nstage=2,ALT_pre=0}	0.014	0.367	0.038	3.089	11
[9]	{Tstage=4,Nstage=2}	0.015	0.364	0.042	3.064	12
[10]	{Tstage=3,HBsAg=1,HBeAg=0,HBeAb=1, EBV=1}	0.014	0.355	0.039	2.990	11
[11]	{Tstage=3,HBsAg=1,HBeAg=0,HBV_DNA_active=0, EBV=1}	0.014	0.355	0.039	2.990	11
[12]	{Tstage=3,HBsAg=1,HBeAb=1, EBV=1}	0.014	0.344	0.040	2.896	11
[13]	{HBsAg=1,HBeAb=1,HBV_DNA_active=0, EBV=1,ALT_pre=0}	0.016	0.342	0.048	2.882	13
[14]	{Tstage=3,HBsAg=1, EBV=1,AST_pre=0}	0.014	0.333	0.042	2.809	11
[15]	{Nstage=1,HBsAg=1,HBeAg=0,HBV_DNA_active=0, EBV=1}	0.014	0.333	0.042	2.809	11
[16]	{Tstage=3,HBsAg=1,HBeAg=0, EBV=1}	0.014	0.324	0.043	2.726	11
[17]	{Nstage=1,HBsAg=1,HBV_DNA_active=0, EBV=1}	0.014	0.324	0.043	2.726	11
[18]	{HBsAg=1,HBeAb=1, EBV=1,ALT_pre=0}	0.016	0.317	0.052	2.672	13
[19]	{Tstage=3,HBsAg=1,HBcAb=1, EBV=1}	0.014	0.314	0.044	2.648	11
[20]	{Tstage=3,HBsAg=1,HBV_DNA_active=0, EBV=1}	0.014	0.314	0.044	2.648	11
**Top 10 rules after exclusion of T/N stage and AST and ALT levels**						
[1]	{HBsAg=1,HBeAg=0,HBeAb=1, EBV=1}	0.018	0.250	0.071	2.106	14
[2]	{HBsAg=1,HBeAg=0, EBV=1}	0.020	0.246	0.082	2.074	16
[3]	{HBsAg=1,HBeAb=1, EBV=1}	0.018	0.246	0.072	2.069	14
[4]	{HBsAg=0,HBeAb=0,HBcAb=1, EBV=1}	0.014	0.239	0.058	2.015	11
[5]	{HBsAg=1, EBV=1}	0.021	0.233	0.092	1.962	17
[6]	{HBsAb=0,HBeAg=0,HBeAb=1 EBV=1}	0.019	0.231	0.082	1.944	15
[7]	{HBsAb=0,HBeAb=1, EBV=1}	0.019	0.227	0.083	1.915	15
[8]	{HBeAg=0,HBeAb=0,HBcAb=1, EBV=1}	0.014	0.224	0.062	1.891	11
[9]	{HBsAb=0,HBeAg=0,HBcAb=1, EBV=1}	0.020	0.216	0.093	1.822	16

© 2023 Li H et al. *JAMA Network Open.*

[10]	{HBeAb=0,HBcAb=1, EBV=1}	0.015	0.214	0.071	1.805	12

Note 1: All rhs were set as distant metastasis; EBV = 1 was set as Plasma EBV DNA load ≥ 1.5 × 1000 copies/mL; HBV_DNA_active = 0 was set as plasma HBV DNA load < 1000 copies/mL or missing data.
Note 2: T stage and N stage were recorded according to the 8th edition AJCC/UICC staging system.
Note 3: All rules were completely repeatable by setting random seed=2020(year this study begin), using R's apriori {arules} package
Abbreviation: Lhs, left hand side; rhs, left hand side; EBV, pretreatment plasma EBV DNA level; HBsAg, hepatitis B surface antigen; HBeAg, hepatitis B e antigen; HBeAb, hepatitis B e antibody; HBcAb, hepatitis B core antibody; ALT, alanine aminotransferase; AST, aspartate transaminase; 1, positive; 0, negative; pre, pretreatment.

© 2023 Li H et al. *JAMA Network Open.*

eTable 2. Test of confounding variables and samples of interaction effect

**Sample of interaction effect**	**Multivariable Cox regression**		
	coef	HR (95%CI)	*P* value
**EBV DNA<br>Cutoff: 1500 copies/mL**			
Stage	0.854	2.35 (1.31–4.21)	.004
HBsAg(+/-)	−1.099	0.33 (0.08–1.41)	.14
EBV DNA	0.543	1.72 (1.05–2.83)	.03
Interaction	1.642	5.17 (1.10–24.21)	.04
**EBV DNA<br>Cutoff: 4000 copies/mL**	Without volume (total data *n* = 792)		
Stage	0.891	2.44 (1.36–4.37)	.003
HBsAg	−1.01	0.36 (0.11–1.19)	.09
EBV DNA	0.419	1.52 (0.95–2.43)	.08
Interaction	1.763	5.83 (1.56–21.78)	.009

Note 1: EBV DNA refers to a larger value vs. a lower value; cutoff value of 1.5 × 1000 copies/mL represented the start point of significant interaction effect; cutoff value of 4 × 1000 copies/mL was selected between 1.5 × 1000 copies/mL to 6 × 1000 copies/mL to show the tendency of interaction effect along the changes in plasma EBV DNA load (**Table 2**). "Stage" here refers to stage III/IV vs. stage I/II and stage I/II as 1 (reference) in the calculation of HR, because when the HBsAg(+) status was used during stratification, the number of patients was considerably low. Thus, using stage I as 1 (reference) would lead to statistical errors, resulting in an inapplicable calculation of HR. Interaction was applicable to both plasma EBV DNA level ≥ larger value and HBsAg(+) vs. other values. This table is a sample of the multivariable analysis (interaction part) presented in **Table 2**, whereby the interaction effect between HBsAg(+) and plasma EBV DNA load was confirmed. A significant interaction effect was confirmed via multivariable Cox regression.

Note 2: Prior to performing multivariable Cox regression analysis, we tested the confounding variables above. From **Table 1**, univariable analysis, stage, EBV, volume, and treatment should be used as confounding variables.

Treatment was evaluated according to the stage based on clinical knowledge; hence, we excluded it when "stage" was considered; volume exhibited a high correlation with stage (0.641) and was also excluded when "stage" was considered.

As we had to analyze the interaction effect, HBsAg was also considered a confounding variable.

In addition, we selected the stage but not according to the T/N classification, as the stage already indicated information in terms of this classification; the results obtained would thus be similar to those obtained with the "stage" mentioned in the above table. However, these results are not presented here.

Therefore, stage, plasma EBV DNA load, and HBsAg were used as confounding variables.

Abbreviations: HBsAg, hepatitis B surface antigen; EBV, Epstein-Barr virus; +, positive; -, negative; coef, coefficient; HR, hazard ratio; CI, confidence interval.

© 2023 Li H et al. *JAMA Network Open.*

eTable 3. Stratification analysis based on HBsAg(+/-) status or different plasma EBV DNA cutoff values

**Stratification analysis**	**Multivariable Cox regression**					
	coef	HR (95%CI)	*P* value	coef	HR (95%CI)	*P* value
**EBV DNA cutoff: 150 copies/mL**	**HBsAg(-) *n* = 651**			**HBsAg(+) *n* = 141**		
Stage	0.757	2.13 (1.15–3.95)	.02	1.575	4.83 (0.64–36.37)	.13
EBV DNA	0.561	1.75 (1.06–2.89)	.03	2.138	8.49 (1.95–36.89)	.004
**EBV DNA cutoff: 4000 copies/mL**	**HBsAg(-) *n* = 651**			**HBsAg(+) *n* = 141**		
Stage	0.892	2.44 (1.32–4.50)	.004	1.330	3.78 (0.50–28.76)	.20
EBV DNA	0.122	1.13 (0.71–1.80)	.61	2.293	9.90 (2.86–34.33)	< .001

Note 1: The cutoff value of 1.5 × 1000 copies/mL represented the start point of significant interaction effect; the cutoff value of 4 × 1000 copies/mL was selected between 1.5 × 1000 copies/mL to 6 × 1000 copies/mL, which was selected to show the tendency of interaction effect along the change of plasma EBV DNA load (**Table 2**).

Note 2: This table is a sample of the multivariable analysis presented in **Table 2** (Step 1. Stratification analysis), which confirmed that the risk (HR) associated with plasma EBV DNA load in the HBsAg(+) group was considerably higher than that in the HBsAg(-) group.

Abbreviations: HBsAg, hepatitis B surface antigen; EBV, Epstein-Barr virus; +, positive; -, negative; coef, coefficient; HR, hazard ratio; CI, confidence interval.

© 2023 Li H et al. *JAMA Network Open.*

eTable 4. Quantification of risk between the HBsAg(+) and HBsAg(-) groups at different plasma EBV DNA loads

EBV DNA level (× 1000 copies/mL)	HBsAg(-)			HBsAg(+)			Comparison	
	N	HR (95%CI)	*P* value	*N*	HR (95%CI)	*P* value	HR (95%CI)	*P* value
= 0[#]	248	1 (reference)		248	1 (reference)		Left as 1 (reference)	
> 0	450	1.94 (1.09–3.47)	.03	94	2.85 (1.39–5.87)	.004	1.44 (0.85–2.44)	.18
> 0.5	398	1.99 (1.11–3.59)	.02	83	3.07 (1.48–6.37)	.003	1.48 (0.86–2.54)	.16
> 1	357	2.14 (1.18–3.86)	.01	76	3.42 (1.65–7.12)	.001	1.55 (0.90–2.67)	.12
> 1.29*	349	2.20 (1.21–3.98)	.009	73	3.61 (1.74–7.49)	.001	1.60 (0.93–2.77)	.09
> 1.5	338	2.16 (1.19–3.93)	.01	71	3.74 (1.80–7.74)	<.001	1.70 (0.98–2.95)	.06
> 2	321	2.21 (1.21–4.04)	.01	67	3.92 (1.90–8.10)	<.001	1.79 (1.03–3.11)	.04
> 3	297	2.28 (1.24–4.22)	.008	58	4.35 (2.08–9.10)	<.001	1.97 (1.11–3.47)	.02
> 4	282	2.29 (1.23–4.26)	.009	56	4.55 (2.18–9.50)	<.001	2.10 (1.18–3.72)	.01
> 5	262	2.12 (1.12–4.01)	.02	52	5.02 (2.39–10.55)	<.001	2.50 (1.39–4.51)	.002
> 6	244	2.04 (1.06–3.90)	.03	50	5.26 (2.47–11.18)	<.001	2.63 (1.45–4.79)	.002
> 7	226	1.82 (0.93–3.55)	.08	46	4.66 (2.14–10.15)	<.001	2.62 (1.38–4.99)	.003
> 8	217	1.90 (0.98–3.70)	.06	45	4.79 (2.20–10.42)	<.001	2.58 (1.35–4.90)	.004
> 9	213	1.94 (1.00–3.77)	.05	43	5.10 (2.33–11.20)	<.001	2.63 (1.38–5.02)	.003
> 10	205	1.97 (1.00–3.86)	.05	43	5.10 (2.33–11.20)	<.001	2.66 (1.39–5.09)	.003
> 20	154	2.01 (0.98–4.13)	.06	31	5.26 (2.19–12.64)	<.001	2.82 (1.31–6.09)	.008
> 30	124	1.97 (0.91–4.23)	.08	28	6.02 (2.51–14.43)	<.001	3.22 (1.45–7.14)	.004
> 40	103	2.11 (0.95–4.66)	.07	21	4.46 (1.63–12.26)	<.001	2.25 (0.86–5.88)	.10
> 50	88	2.29 (1.02–5.12)	.04	16	3.86 (1.19–12.53)	.02	1.81 (0.58–5.70)	.30

[#]By setting plasma EBV DNA load =0 as the same reference group in both HBsAg(+/-) groups, the HRs in the two groups were made comparable.

*1.29 × 1000 copies/mL was the most appropriate cutoff value to demonstrate interaction effect occurring via the HBsAg(-) group, as presented in **eFigure 2 in Supplement.**

Note 1: The metastasis risk (HR) in the HBsAg(+) group increased gradually with the increase in the plasma EBV DNA load, whereas the same pattern was not observed in the HBsAg(-) group.

Note 2: When the plasma EBV DNA load ranging from 2 × 1000 copies/mL to 30 × 1000 copies/mL, the metastasis risk (HR) in the HBsAg(+) group was always higher than that in the HBsAg(-) group at the same plasma EBV DNA load (*P* <.05).

Note 3: The above findings were also supported by and could be visualized using the RCS and Kaplan–Meier survival curves **(Figure 2)**.

Abbreviations: HBsAg, hepatitis B surface antigen; EBV, Epstein–Barr virus; +, positive; -, negative; HR, hazard ratio; CI, confidence interval.

© 2023 Li H et al. *JAMA Network Open.*
