# Supplementary material for: Synergistic Association of Hepatitis B Surface Antigen and Plasma Epstein-Barr Virus DNA Load on Distant Metastasis in Patients With Nasopharyngeal Carcinoma
Source: JAMA Netw Open. 2023 Feb 9;6(2):e2253832. doi: 10.1001/jamanetworkopen.2022.53832 (PMC9912125; doi:10.1001/jamanetworkopen.2022.53832)
Supplement: Supplement 2. — Data Sharing Statement [file jamanetwopen-e2253832-s002.pdf]

Data Sharing Statement

Li. Synergistic Association of Hepatitis B Surface Antigen and Plasma Epstein-Barr Virus DNA Load on Distant Metastasis in Patients With Nasopharyngeal Carcinoma. *JAMA Netw Open*. Published February 09, 2023. doi:10.1001/jamanetworkopen.2022.53832

Data
Data available: Yes
Data types: Participant data with identifiers, Data (not involving human participants), Data dictionary
How to access data: The authenticity of the study was validated by uploading the fully raw data onto the Research Data Deposit (RDD) public platform (http://www.researchdata.org.cn). The data that support the findings of this study are available from the corresponding author upon reasonable request.
When available: With publication

Supporting Documents
Document types: None

Additional Information
Who can access the data: Researchers whose proposed use of the data has been approved.
Types of analyses: Retrospective study and survival analysis.
Mechanisms of data availability: After approval of a proposal, or with a signed data access agreement.
Any additional restrictions: No commercial study.
